# Pericoronary adipose tissue inflammation mediates the atherogenic effects of lipids on multivessel coronary artery disease: a CCTA-based radiomics analysis

**DOI:** 10.3389/fcvm.2025.1629984

**Published:** 2025-09-24

**Authors:** Haimei Du, Junchen Zheng, Yaxin Yao, Qin Zhou, Linjuan Li

**Affiliations:** ^1^ Department of General Medicine, Yan’an University Affiliated Hospital, Yan'an, Shaanxi, China; ^2^ Department of Cardiovascular Medicine, Yan’an University Affiliated Hospital, Yan'an, Shaanxi, China; ^3^ Yan'an University Affiliated Hospital, Yan'an, Shaanxi, China

**Keywords:** atherogenic index of plasma, coronary atherosclerotic disease severity, coronary arterial inflammation, glucose metabolism status, mediation analysis

## Abstract

**Objective:**

The atherogenic index of plasma (AIP) is a robust predictor of cardiovascular risk. However, its mechanism of action in the severity of coronary artery disease (CAD) remains unknown. We investigated whether pericoronary adipose tissue inflammation [assessed using the fat attenuation index (FAI)] mediates the association between AIP and CAD in middle-aged and older adults.

**Methods:**

A total of 450 patients who underwent coronary computed tomography angiography at Yan'an University Affiliated Hospital (2022–2024) were enrolled in this study. Coronary atherosclerotic disease (CAD) severity was defined as multivessel CAD (MVCAD; ≥50% stenosis in ≥2 arteries). The fat attenuation index (FAI) was measured around the right coronary artery (RCA-FAI) using a standardized radiomics protocol. Logistic regression and mediation analyses (PROCESS macro, 1,000 bootstrap samples) were used to quantify these associations.

**Results:**

The atherogenic index of plasma (AIP) independently predicted MVCAD (OR = 2.35, 95% CI: 1.96–5.10, *P* < 0.01). The RCA-FAI showed a dose-dependent CAD risk (OR = 1.33 per one-unit increase, *P* < 0.01), with a 33% higher risk per FAI increment. Mediation analysis revealed that the RCA-FAI explained 27.9% of the AIP–MVCAD association (*P* < 0.05). Stratification by glucose metabolism status confirmed the consistent role of the RCA-FAI across subgroups, whereas the AIP–CAD association was significant only in normoglycemic individuals.

**Conclusion:**

This is the first study to demonstrate that coronary arterial inflammation (RCA-FAI) partially mediates the atherogenic effects of AIP on MVCAD, suggesting a dual pathway of lipid-driven inflammation and metabolic dysregulation. Our findings highlight RCA-FAI as a promising imaging biomarker for CAD risk stratification, irrespective of glucose metabolism status.

## Background

With the ongoing global demographic aging and lifestyle modifications, the incidence of coronary atherosclerotic disease (CAD) continues to rise worldwide, solidifying its position as a leading cause of mortality and disability (1). As a multifactorial disorder, CAD profoundly impacts the quality of life of patients while imposing a substantial socioeconomic burden on families and healthcare systems. These realities underscore the critical importance of early diagnostic stratification and timely therapeutic intervention.

The atherogenic index of plasma (AIP), calculated from the logarithmic ratio of triglycerides (TG) to high-density lipoprotein cholesterol (TG/HDL-C), has emerged as a novel biomarker for assessing subclinical atherosclerosis and cardiovascular risk stratification (2). Accumulating evidence demonstrates significant correlations between elevated AIP and coronary atherosclerotic disease (CAD) severity, particularly in patients with normal glucose regulation (NGR) (3). Notably, AIP serves as an independent prognostic factor in CAD populations, with longitudinal studies linking cumulative AIP exposure to increased risks of major adverse cardiovascular events (MACE), stroke, and myocardial infarction, particularly among elderly cohorts (4, 5). Analysis of the National Health and Nutrition Examination Survey (NHANES) 20052018 data further corroborates AIP's predictive capacity for future MACE and cardiac mortality (6).

In contemporary CAD management, coronary computed tomography angiography (CCTA) has become indispensable for evaluating chronic coronary syndromes (7). The fat attenuation index (FAI) and pericoronary adipose tissue (PCAT) volumequantitative parameters derived from CCTAprovide non-invasive biomarkers of coronary inflammation and atherosclerotic burden (8, 9). Specifically, right coronary artery (RCA) FAI and total PCAT volume have been validated as independent predictors of CAD prevalence and progression (10). A recent multicenter longitudinal cohort study of 40,091 CCTA-examined patients [median follow-up: 2.7 years (IQR 1.4–5.3)] demonstrated significant associations between FAI scores and risks of cardiac mortality/MACE, highlighting its prognostic utility even in non-obstructive CAD (6).

Despite these advances, the mechanistic interplay between AIP, coronary inflammation (as quantified by FAI/PCAT), and CAD severity remains incompletely characterized. No prior study has systematically investigated whether coronary inflammatory activity mediates the relationship between atherogenic lipid profiles and multivessel CAD progression in aging populations. This study, therefore, aims to elucidate the association between AIP and CAD severity in middle-aged and elderly patients, with particular emphasis on the mediating role of RCA-FAI assessed coronary inflammation.

## Methods

### Research population

**Figure 1:**
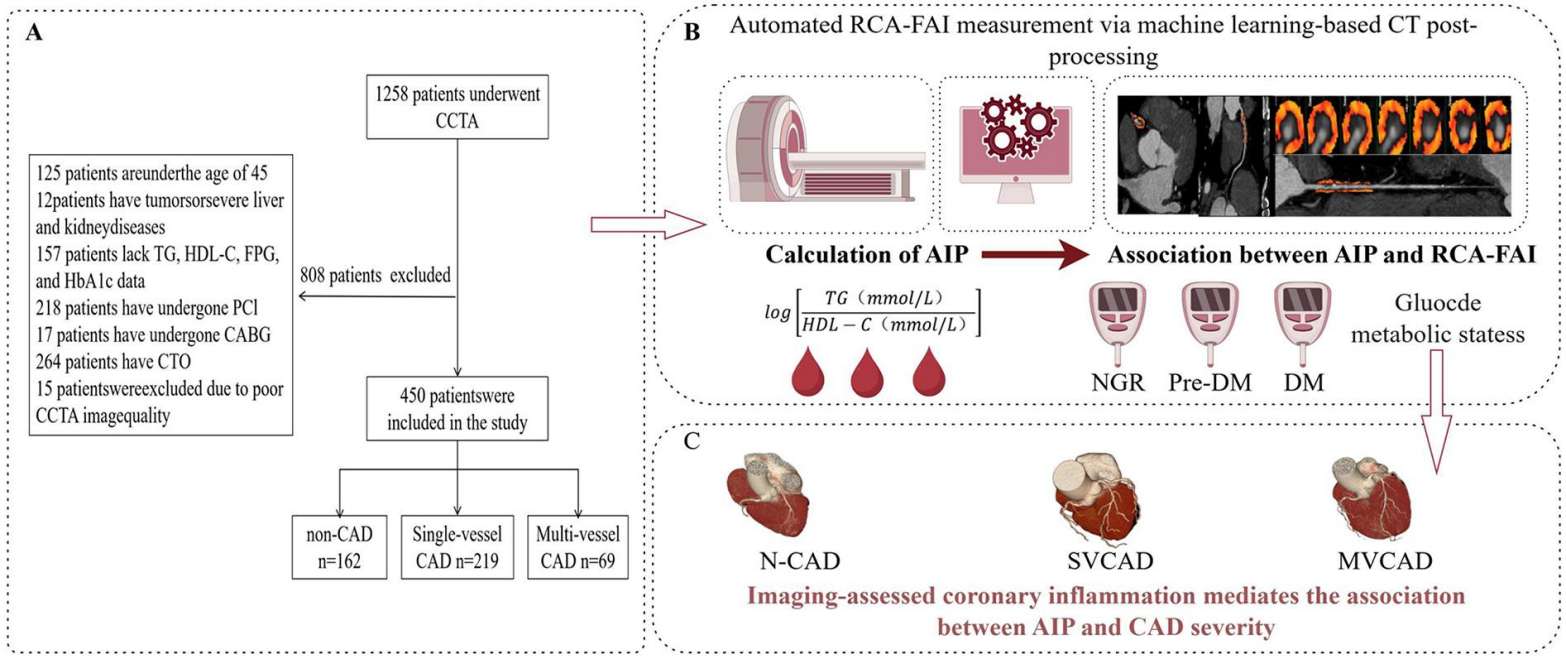
Flowchart of patient recruitment and study design. (**A)** Explained patient inclusion/exclusion criteria and cohort stratification (main finding: 450 patients included, grouped by CAD severity). **(B)** Describes the automated RCA-FAI measurement, AIP calculation, and the link between systemic (AIP) and RCA-FAI. **(C)** Explicitly stated the mechanistic hypothesis (coronary inflammation mediates AIP–CAD severity) and illustrated group differences. CCTA, coronary computed tomography angioraphy; TG, triglyceride; HDL-C, high-density lipoprotein chlolesterol; FPG, fasting plasma glucose; HbAlc, glycated hemoglobin; PCI, percutaneous coronary intervention; CABG, coronary artery bypass grafting; CTO, chronic total occlusion; AIP, atherosclerosis index of plasma; RCA-FAI, right coronary artery perivascular fat attenuation index; non-coronary artery disease; SVCAD, single-vessel coronary artery disease; NGR, normal glucose regulation; Pre-DM, prediabetes mellitus; DM, diabetis mellitus; N-CAD, non-coronary artery disease; SVCAD, single-vessel coronary artery disease; MVCAD, multivessel coronary artery disease; CAD, coronary artery disease. Created in figdraw.com.

This investigation initially screened 1,258 consecutive patients who underwent CCTA at Yan'an University Affiliated Hospital between January 2022 and August 2024. The exclusion criteria comprised (1) age <45 years; (2) presence of malignancies or severe hepatic/renal dysfunction; (3) missing laboratory data including TG, HDL-C, fasting plasma glucose (FPG), and glycated hemoglobin (HbA1c); (4) prior revascularization via percutaneous coronary intervention (PCI) or coronary artery bypass grafting (CABG); (5) chronic total coronary occlusion (CTO); and (6) suboptimal CCTA image quality caused by respiratory motion artifacts or cardiac arrhythmia. After rigorous screening, 450 eligible patients were ultimately enrolled. Figure 1 illustrates the participant recruitment process and study design framework.

### Data collection

Demographic, anthropometric, and clinical data were extracted from electronic health records. The key anthropometric parameters included age, sex, height, weight, and body mass index [BMI; weight (kg)/height (m)^2^]. Clinical history included hypertension, diabetes mellitus (DM), and smoking status.

Medications: Glucose-lowering agents, statins, aspirin, and fasting (>8 h) venous blood samples were analyzed for FPG (hexokinase method), lipid profile (TG), total cholesterol, low-density lipoprotein cholesterol (LDL-C), HDL-C (enzymatic colorimetric assays), HbA1c (high-performance liquid chromatography), and serum creatinine (modified Jaffe method). The AIP was calculated as follows: AIP = log10[TG (mmol/L) / HDL-C (mmol/L)]. CAD severity assessment: Two blinded cardiovascular radiologists independently evaluated CCTA images using standardized criteria—CAD diagnosis, ≥50% stenosis in ≥1 major coronary artery; MVCAD classification, ≥50% stenosis in ≥2 major coronary arteries (11).Glucose metabolism stratification [per American Diabetes Association (ADA) guidelines]: DM, FPG ≥7.0 mmol/L, 2h-PG ≥11.1 mmol/L, HbA1c ≥6.5%, or current hypoglycemic therapy; prediabetes, FPG 5.6–6.9 mmol/L, 2h-PG 7.8–11.0 mmol/L, or HbA1c 5.7%–6.4%. NGR was defined as FPG <5.6 mmol/L and HbA1c <5.7% (12). Hypertension was defined as a systolic blood pressure (BP) of ≥140 mmHg, a diastolic BP of ≥90 mmHg, or use of antihypertensive treatment.

### CCTA imaging protocol

All scans were performed using a 256-slice dual-source CT scanner (SOMATOM Definition Flash; Siemens Healthcare, Munich, Germany) with retrospective ECG gating. The technical parameters were as follows: tube voltage 120 kV and tube current 250–800 mA (modulated by the automatic exposure control), collimation 128 × 0.625 mm, gantry rotation 270 ms, and reconstruction 0.75 mm slice thickness, with 0.5 mm increment. Patients received β-blockers (25–75 mg oral metoprolol) if their baseline heart rate exceeded 70 bpm. Contrast protocol: iodixanol (320 mg I/mL; 60–80 mL at 4.5–6.5 mL/s) via antecubital vein. Adipose tissue was defined as all voxels in the Hounsfield units (HU) range between −190 and −30 HU located within a radial distance from the outer vessel border equal to the diameter of the surrounding vessel (CT-FFR V1.7, FAI V1.2, ShuKun Technology Co., Ltd., Beijing, China) (11, 13). Correlations between the peripheral coronary FAI based on computed tomography images, high-risk plaque, and degree of coronary artery stenosis in patients with coronary atherosclerosis (14). Key processing steps included segmentation of proximal coronary segments [left anterior descending coronary artery (LAD), from the ostium to 40 mm distal; left circumflex coronary artery(LCX), from the ostium to 40 mm distal; and RCA, 10–50 mm from the ostium], automated PCAT volume quantification within a 3 mm radial distance from the vessel wall, and FAI calculation as the mean HU value of PCAT.

### Statistical analysis

In this study, data normality was assessed using the Shapiro–Wilk test. Continuous variables with normal distribution were summarized as mean ± standard deviation (SD), whereas non-normally distributed variables were reported as median with IQR. Categorical variables were expressed as frequencies (percentages). For group two-group comparisons, Student's *t*-test was used for normally distributed data, while the Mann–Whitney *U* test was used for non-normally distributed data. For multi-group comparisons, one-way ANOVA was used for normally distributed data, while the Kruskal–Wallis test was used for non-normally distributed data. Multivariable logistic regression was used to analyze associations between AIP, RCA-FAI, and MVCAD. Mediation effects were quantified using Hayes' PROCESS macro (Model 4) in SPSS 26.0, with AIP as the independent variable (continuous), RCA-FAI (continuous) as the mediator, and MVCAD (binary) as the dependent variable (10, 15, 16). This study used a directed acyclic graph to visualize the assumed causal model with AIP (continuous) as the exposure, RCA-FAI (continuous) as the mediator, and multivessel CAD as the outcome variable. Confounders identified using directed acyclic graphs were adjusted for. The significance of the mediating effect was examined using 1,000 bootstrap samples, with statistical significance defined as two-tailed *P* < 0.05.

## Results

### Baseline characteristics

The study cohort comprised 450 patients [mean age, 63.72 ± 9.07 years; male predominance (53.8%)]. The key biomarkers included an average AIP of 0.50 ± 0.26, mean RCA-FAI of −83.48 ± 8.04 HU, and median RCA-PCAT volume of 1,381.74 mm^3^. Patients with MVCAD were significantly older and had higher systolic/diastolic blood pressure and hypertension prevalence than those in the single-vessel CAD (SVCAD) and non-coronary atherosclerotic disease (N-CAD) groups (*P* < 0.05). Notably, the prevalence of prediabetes/diabetes and the use of glucose-lowering agents/statins were higher in the SVCAD and MVCAD subgroups than that in the N-CAD controls (*P* < 0.01). Comparative analysis revealed that patients with RCA-FAI had lower values (−88.64 ± 6.90 HU vs. SVCAD −83.48 ± 2.30 HU vs. N-CAD −73.77 ± 5.26 HU, *P* < 0.01) and reduced RCA-PCAT volumes (*P* < 0.001). No intergroup differences were observed in sex, BMI, smoking status, FPG, HbA1c, LAD/left circumflex coronary artery (LCX) artery FAI values, LAD/LCX-PCAT volumes, or aspirin use (all *P* > 0.05) (Table 1).

**Table 1:** Demographic and clinical characteristics.

Characteristics	Total patients	N-CAD	SVCAD	MVCAD	*F*/*H*/*χ*^2^	*P* value
*N*	450	162	219	69		
Age, years	63.72 ± 9.07	62.64 ± 8.76	63.54 ± 9.09	66.84 ± 9.17^a^^,^^b^	*F* = 3.14	0.01
Male, *n* (%)	242 (53.8)	94 (58.0)	108 (19.3)	40 (58.0)	*χ*^2^ = 2.73	0.18
BMI, kg/m^2^	24.92 ± 3.33	25.04 ± 3.48	24.86 ± 3.29	24.80 ± 3.11	*F* = 1.62	0.83
SBP, mmHg	140.19 ± 17.01	138.70 ± 16.14	139.74 ± 17.3	145.77 ± 17.44^c^^,^^d^	*F* = 1.53	0.03
DBP, mmHg	85.34 ± 11.49	84.7 ± 11.55	84.71 ± 11.11	88.86 ± 12.1^c^^,^^d^	*F* = 0.84	0.02
Smoking, *n* (%)	171 (38)	60 (37)	81 (37)	30 (43.5)	*χ*^2^ = 0.45	0.61
Hypertension, *n* (%)	319 (70.89)	113 (69.8)	155 (70.8)	51 (73.9)^a^	*χ*2 = 0.40	<0.01
Glucose metabolic states, *n* (%)					*χ*^2^ = 4.25	<0.01
NGR, *n* (%)	229 (50.9)	91 (56.2)	104 (47.5)^a^	34 (49.3)^a^^,^^b^		
Pre-DM, *n* (%)	130 (28.9)	40 (24.7)	69 (31.5)^a^	21 (30.4)^a^		
DM, *n* (%)	91 (20.2)	31 (19.1)	46 (21)^c^	14 (20.3)^c^		
Serum biomarkers						
FPG, mmol/L	6.33 ± 2.04	6.06 ± 1.7	6.27 ± 1.88	6.52 ± 2.34	*F* = 0.97	0.24
HbA1c, %	6.48 ± 1.38	6.37 ± 1.21	6.4 ± 1.25	6.64 ± 1.58	*F* = 0.33	0.22
Triglyceride, mmol/L	1.98 ± 1.29	1.52 ± 0.67	2.41 ± 1.56^c^	2.7 ± 0.94^c^^,^^d^	*F* = 141.54	0.01
Total cholesterol, mmol/L	4.21 ± 1.12	4.13 ± 1.13	4.37 ± 1.11^c^	4.92 ± 1.05^b^^,^^c^	*F* = 10.01	<0.01
HDL-C, mmol/L	1.07 ± 0.24	1.14 ± 0.24	1.01 ± 0.22^c^	0.76 ± 0.20^b^^,^^c^	*F* = 85.47	<0.01
LDL-C, mmol/L	2.11 ± 0.8	2.06 ± 0.81	2.21 ± 0.82^c^	2.89 ± 0.74^c^^,^^d^	*F* = 11.90	0.01
AIP	0.50 ± 0.26	0.31 ± 0.22	0.50 ± 0.13^c^	0.82 ± 0.14^a^^,^^b^	*F* = 129.03	<0.01
FAI, HU						
RCA	−83.48 ± 8.04	−88.64 ± .6.90	−83.48 ± 2.30^c^	−73.77 ± 5.26^c^^,^^d^	*F* = 0.57	0.01
LAD	−79.61 ± 7.96	−79.68 ± 7.07	−79.7 ± 8.44	−79.17 ± 8.45	*F* = 1.542	0.88
LCX	−71.47 ± 8.02	−71.15 ± 7.84	−71.767 ± 8.1	−71.28 ± 8.25	*F* = 0.282	0.75
PCAT volume, mm^3^						
RCA	1,381.74 (931.66 (1,936.35)	1,442.395 (1,010.59 (1,994.98)	1,407.38 (1,002.64 (2,000.22)	948.16 (639.08 (1,667.515)^c^^,^^d^	*H* = 1.532	<0.01
LAD	1,166.17 (837.40 (1,588.15)	1,185.66 (899.74 (1,576.38)	1,150.35 (821.47 (1,632.2)	1,065.04 (632.1 (1,551.92)	*H* = 2.963	0.33
LCX	505.36 (327.60 (782.58)	522.02 (333.01 (805.22)	510.74 (330.87 (772.1)	457.42 (274.57 (832.08)	*H* = 0.865	0.46
Medications, *n* (%)						
Antidiabetic drugs, *n* (%)	82 (18.2)	26 (16.0)	42 (19.2)^c^	14 (20.3)^a^^,^^b^	*χ*^2^ = 0.769	<0.01
Statin, *n* (%)	350 (77.8)	117 (72.28)	175 (79.9)^c^	58 (84.1)^a^^,^^b^	*χ*^2^ = 1.915	<0.01
Aspirins, *n* (%)	272 (60.4)	97 (59.9)	132 (60.3)	43 (62.3)	*χ*^2^ = 2.004	0.78

N-CAD, non- coronary artery disease; SVCAD, single-vessel coronary artery disease; MVCAD, multivessel coronary artery disease; BMI, body mass index; SBP, systolic blood pressure; DBP, diastolic blood pressure; NGR, normal glucose regulation; Pre-DM, prediabetes mellitus; DM, diabetes mellitus; FPG, fasting plasma glucose; HDL-C, high-density lipoprotein cholesterol; LDL-C, low-density lipoprotein cholesterol; AIP, atherosclerosis index of plasma; FAI, fat attenuation index; RCA, right coronary artery; LAD, left anterior descending coronary artery; LCX, left circumflex coronary artery; PCAT, pericoronary adipose tissue.

*F*, *F*-statistic from one-way ANOVA (for normally distributed continuous variables); *H*, *H*-statistic from Kruskal–Wallis test (for non-normally distributed continuous variables); *χ*^2^, chi-square statistic (for categorical variables). Values are presented as mean ± SD or median (IQR) for continuous variables and *n* (%) for categorical variables.

^a^ Adjusted *P* < 0.01 vs. N-CAD.

^c^ Adjusted *P* < 0.05 vs. N-CAD.

^b^ Adjusted *P* < 0.01 vs. SVCAD.

^d^ Adjusted *P* < 0.05 vs. SVCAD.

### Association of AIP with PCAT volume and FAI

Stratifying AIP into tertiles (Q1, ≤0.38; Q2, 0.39–0.61; Q3, ≥0.62) revealed progressive RCA-FAI elevation (Q1 −86.93 ± 7.19 HU vs. Q3 −78.93 ± 7.19 HU, *F* = 7.96, *P* < 0.001) and RCA-PCAT volume reduction (Q1 2,217.75 ± 786.05 mm^3^ vs. Q3 1,316.73 ± 860.53 mm^3^, *F* = 7.754, *P* < 0.001). LAD-PCAT volumes showed modest tertile differences (Q1 1,372.51 ± 718.52 mm^3^ vs. Q3 1,150.08 ± 560.59 mm^3^, *F* = 2.232, *P* = 0.032). No significant differences were observed in the FAI measurements of the LAD, LCX, or LCX-PCAT volumes (all *P* > 0.05). The complete data are listed in Table 2.

**Table 2:** Characteristics of FAI and PCAT volume in participants measuring plasma atherosclerosis index (in tertiles).

Characteristics	Q1	Q2	Q3	*F*	*P* value
	AIP ≤ 0.38	0.39 < AIP ≤ 0.61	AIP ≥ 0.62		
*N*	150	150	150		
FAI, HU					
RCA	−81.06 ± 8.845	−82.08 ± 7.94^a^	−82.30 ± 7.06^b^	7.96	<0.01
LAD	−78.49 ± 8.54	−79.22 ± 6.97	−80.36 ± 8.24	0.13	0.20
LCX	−71.35 ± 8.98	−71.02 ± 7.51	−71.53 ± 7.83	0.29	0.84
PCAT volume, mm^3^					
RCA	528.996 ± 36.16	786.05 ± 52.76^b^	823.19 ± 32.54^b^^,^^c^	7.75	<0.01
LAD	1,150.08 ± 560.59	1,247.74 ± 573.45	1,372.51 ± 718.52^a^	2.23	0.03
LCX	679.85 ± 787.49	608.21 ± 402.82	574.84 ± 345.09	1.55	0.46

FAI, fat attenuation index; RCA, right coronary artery; LAD, left anterior descending coronary artery; LCX, left circumflex coronary artery; PCAT, pericoronary adipose tissue.

*F*, *F*-statistic from one-way ANOVA (for normally distributed continuous variables).

^a^ Adjusted *P* < 0.05 vs. Q1 (low AIP).

^b^ Adjusted *P* < 0.01 vs. Q1.

^c^ Adjusted *P* < 0.05 vs. Q2 (moderate AIP).

### Association of AIP and RCA-FAI with CAD severity

Logistic regression analysis demonstrated a borderline association between AIP and MVCAD in the unadjusted models (OR = 1.76, 95% CI: 1.68–2.19, *P* *<* 0.01). Adjusting for age and sex strengthened this association (Model 2: OR = 1.79, 95% CI: 1.69–2.35, *P* < 0.01), which persisted after additional adjustments for BMI, blood pressure, smoking, hypertension, and medication use (Model 3: OR = 2.35, 95% CI: 1.96–5.10, *P* < 0.01). The RCA-FAI consistently predicted MVCAD across all models (*P* *<* 0.01), with effect sizes increasing from Model 1 (OR = 1.30) to Model 3 (OR = 1.33), indicating a 33% increase in MVCAD risk per one-unit RCA-FAI increment (Table 3).

**Table 3:** Logistic regression analysis of the correlation between AIP, RCA-FAI, and the severity of CAD.

Characteristics	Model 1		Model 2		Model 3	
	OR (95%CI)	*P* value	OR (95%CI)	*P* value	OR (95%CI)	*P* value
AIP	1.76 (1.68–2.19)	<0.01	1.79 (1.69–2.35)	<0.01	2.35 (1.96–5.10)	<0.01
RCA-FAI	1.30 (1.10–1.50)	<0.01	1.30 (1.10–1.49)	<0.01	1.33 (1.12–1.52)	<0.01

Model 1, unadjusted.

Model 2, adjusted for age and sex.

Model 3, adjusted for sex, age, BMI, systolic blood pressure, diastolic blood pressure, smoking, hypertension, use of antidiabetic medication, use of antiplatelet medication, and use of lipid-lowering medication.

AIP, atherosclerosis index of plasma; RCA-FAI, right coronary artery perivascular fat attenuation index; CAD, coronary artery disease; CI, confidence interval; BMI, body mass index.

### Glucose metabolism stratification

As shown in Table 4, the AIP was correlated with MVCAD severity exclusively in normoglycemic (NGR) patients (*P* < 0.01). Conversely, the RCA-FAI maintained robust associations with CAD severity across all glucose metabolism subgroups (*P* < 0.05), irrespective of glycemic status.

**Table 4:** Relationship between AIP and RCA-FAI with MVCAD under different glucose metabolism states.

Glucose metabolic states	Model 1		Model 2		Model 3	
	OR (95%CI)	*P* value	OR (95%CI)	*P* value	OR (95%CI)	*P* value
NGR						
AIP	1.89 (1.31–3.21)	0.01	2.19 (1.31–3.98)	0.01	3.05 (1.36–4.20)	<0.01
RCA-FAI	1.72 (1.47–2.02)	<0.01	1.77 (1.49–2.10)	<0.01	4.08 (1.75–12.55)	<0.01
Pre-DM						
AIP	2.60 (1.54–7.07)	0.03	2.19 (1.21–7.80)	0.05	3.57 (2.09–9.45)	0.08
RCA-FAI	1.87 (1.45–2.41)	<0.01	1.86 (1.45–2.39)	<0.01	3.47 (2.63–12.06)	0.01
DM						
AIP	1.49 (0.30–5.49)	0.13	1.42 (1.28–5.79)	0.06	3.36 (2.04–9.51)	0.08
RCA-FAI	2.25 (1.93–5.10)	<0.01	3.41 (2.07–5.63)	0.01	5.02 (1.94–12.97)	0.03

Model 1, unadjusted.

Model 2, adjusted for age and sex.

Model 3, adjusted for sex, age, BMI, smoking, hypertension, use of antidiabetic medication, use of antiplatelet medication, and use of lipid-lowering medication.

AIP, atherosclerosis index of plasma; RCA-FAI, right coronary artery perivascular fat attenuation index; MVCAD, multivessel coronary artery disease; CI, confidence interval; BMI, body mass index.

### Mediation analysis

**Figure 2:**
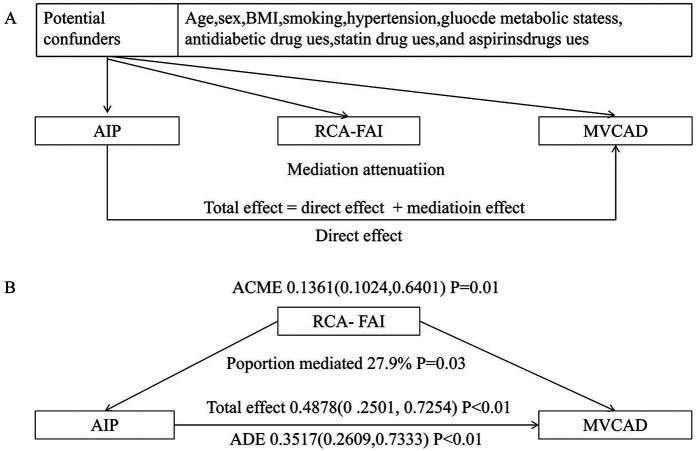
Mediation effect model diagram. **(A)** Directed acyclic graph; **(B)** mediation analysis of MVCAD. MVCAD, multivessel coronary artery disease; ACME, average causal mediation effect; ADE, average direct effect; RCA-FAI, right coronary artery perivascular fat attenuation index; AIP, atherosclerosis index of plasma; BMI, body mass index.

Path analysis revealed a significant total effect of AIP on MVCAD (*β* = 0.49, 95% CI: 0.25–0.73, *P* < 0.01). This association comprised both direct effects (*β* = 0.35, 72.1% proportion mediated) and RCA-FAI-mediated indirect effects (*β* = 0.14, 27.9% mediation proportion), confirming partial mediation by coronary inflammation (Figure 2).

## Discussion

This study investigated the interplay between coronary arterial inflammation, AIP, and CAD severity across various glucose metabolism subtypes in middle-aged and elderly Chinese individuals. Mediation analysis revealed that the RCA-FAI played a partial mediating role (27.9%) in the association between AIP and CAD severity, highlighting the critical role of the “lipid-inflammation signaling pathway” in the development of CAD in middle-aged and older individuals. This result validates existing evidence supporting AIP as an independent predictor of CAD severity, which is consistent with the study by Wang and He (17).

Recent studies have shown that AIP can serve as an independent predictor of cardiovascular risk, and its ability to predict the progression of atherosclerotic plaques and the incidence of CAD is significantly superior to that of traditional risk factors, such as hypertension and diabetes (18). From a mechanistic perspective, AIP reflects the dynamic balance between small dense low-density lipoprotein cholesterol (sdLDL-C) and anti-atherosclerotic lipoproteins and serves as a practical surrogate marker for sdLDL-C levels (19). sdLDL-C is characterized by a smaller particle size and higher density and can accelerate atherosclerosis through the following pathways: (1) reduced particle size (18–22 nm vs. 22–25 nm for LDL-C) increases endothelial permeability; (2) lower sialic acid content promotes binding to proteoglycans, prolonging arterial retention time; (3) decreased affinity for LDL receptors leads to prolonged circulating half-life; (4) sdLDL-C is easily oxidized into pro-inflammatory oxidized low-density lipoprotein (oxLDL); (5) sdLDL-C stimulates macrophages to secrete cytokines and foam cells (20–23).

The 2023 “Chinese Guidelines for Blood Lipid Management” list small dense sdLDL-C as a key biomarker for risk stratification of atherosclerotic cardiovascular disease (ASCVD) (24). Multiple cohort studies have confirmed that sdLDL-C levels are significantly associated with the risk of developing coronary heart disease and its clinical outcomes (25–28). However, the detection of sdLDL-C has a high technical threshold and is costly, making it difficult to promote its application in routine clinical practice (29). In contrast, AIP is derived from routinely measured clinical lipid parameters. This approach incurs negligible additional costs when establishing AIP as a clinically reliable surrogate marker of sdLDL-C (19).

A meta-analysis showed that elevated AIP levels were independently associated with CAD, regardless of whether continuous or categorical variables were used (4, 18). However, this association exhibited heterogeneity across different glucose metabolic states. Wu et al. (3) found a significant positive correlation between AIP and CAD severity in individuals with normal glucose tolerance (NGR), which is consistent with the results of this study; however, in subgroups with abnormal glucose metabolism (such as prediabetes and diabetes), this association was weakened. This heterogeneity may be related to pathways mediated by insulin resistance, which can intensify lipid metabolic disorders by increasing the release of free fatty acids and inhibiting lipoprotein lipase activity, thereby weakening the atherogenic effect of AIP (30, 31). In addition, this study found that even among patients with diabetes, in whom the association between AIP and CAD was weakened, RCA-FAI was still significantly associated with MVCAD, suggesting that inflammation may play a key role in severe CAD, independent of lipid dysregulation and DM.

This study found that RCA-FAI was significantly and positively correlated with CAD severity, consistent with previous studies (10). This result supports the view that “coronary artery inflammation is a core mechanism in the pathophysiology of CAD” (32). When the inflammatory cascade is activated, an elevated RCA-FAI indicates chronic low-grade inflammation, which promotes atherosclerosis progression through the following pathways: leukocyte infiltration, macrophage infiltration, and T-lymphocyte infiltration into the subendothelial layer, leading to lipid deposition and foam cell formation, vascular remodeling, activation of matrix metalloproteinases (MMP-2/9) that degrade the extracellular matrix resulting in vascular dilation or stenosis (33, 34), and release of pro-inflammatory factors, such as tumor necrosis factor-α (TNF-α), interleukin-6 (IL-6), and monocyte chemoattractant protein-1 (MCP-1), accelerating plaque formation (35).

Although RCA-FAI was strongly associated with MVCAD severity, no significant association was observed between LAD-FAI, LCX-FAI, and MVCAD severity. This is consistent with the current consensus that “the RCA is the optimal anatomical site for assessing coronary artery inflammation” (36, 37). In addition, the anatomical stability of the RCA-FAI (located within the right atrioventricular groove with minimal cardiac movement) makes its measurement more reproducible than that of LAD-FAI and LCX-FAI (38). The purpose of including LAD-FAI and LCX-FAI in this study was to explore whether “multivessel FAI assessment can enhance risk stratification more than using RCA-FAI alone.” However, the results showed that LAD-FAI and LCX-FAI were not significantly associated with MVCAD, further confirming the primary role of RCA-FAI as a biomarker of coronary inflammation in the study population.

Stratified analysis of glucose metabolism showed that RCA-FAI was significantly associated with CAD severity in subgroups with normal blood glucose levels, prediabetes, and diabetes, consistent with the results of a cross-sectional cohort study involving 207 patients, which found that both RCA-FAI and total PCAT volume were independent predictors of coronary artery disease (10). In addition, among patients with stable angina, combining FAI with plaque burden indicators (such as plaque volume) can significantly improve the accuracy of ischemic lesion detection (AUC = 0.821) (39). Baseline quantification of perivascular inflammation by FAI is also significantly associated with the incidence of in-stent restenosis during follow-up (40). Unlike previous studies, which primarily used the “incidence of cardiovascular events” as the main endpoint, this study adopted validated threshold criteria (≥50% stenosis in two or more major coronary arteries) to quantify the severity of CAD, focusing on the anatomical severity of lesions. This design helped us directly explore the relationship between inflammation and CAD lesion progression.

AIP is a validated biomarker of sdLDL-C levels and is mechanistically linked to the activation of inflammatory cascades. We hypothesized that coronary inflammation mediates the association between AIP and CAD severity. This study used mediation analysis to investigate whether coronary inflammation (quantified by FAI) mediates the relationship between AIP (assessing lipoprotein abnormalities) and MVCAD. The analysis showed that the FAI mediated 27.9% of the association between AIP and MVCAD, suggesting that inflammation in the PCAT may be involved in the development of this pathological pathway. These results support the hypothesis proposed by Antonopoulos et al. (8) that inflammation of the perivascular adipose tissue is a key driver of metabolically related atherosclerotic disorders.

However, the 27.9% partial mediation result indicates that, in addition to FAI-mediated inflammation, other pathophysiological mechanisms are involved in the development of AIP-related MVCAD. This finding is consistent with the concept proposed by Wolf and Ley (41) that “atherosclerosis is a systemic disease. Although our mediation model statistically supports the inflammation-driven pathway of “AIP→RCA-FAI→MVCAD,” the possibility of bidirectional interactions is worth exploring based on established pathophysiological mechanisms. (1) Lipid-driven pathway: sdLDL-C retention and oxidation activate endothelial LOX-1 receptors, triggering NF-κB signalling and pro-inflammatory cytokine expression (35, 42–44). This cascade response is consistent with our observations, in which higher AIP quartiles were associated with progressively higher RCA-FAI. (2) Inflammation-driven pathway: Chronic vascular inflammation dysregulates lipid metabolism via pro-inflammatory cytokines (e.g., TNF-α and IL-6), suppresses lipoprotein lipase, inhibits HDL biosynthesis, and stimulates very low-density lipoprotein production, thereby elevating the TG/HDL-C ratio (45–47). Adipose tissue inflammation further aggravates lipid metabolic disorders by increasing free fatty acid flux and inducing insulin resistance (48). Studies have shown that chronic inflammatory states (such as chronic periodontitis and chronic hidradenitis suppurativa) are associated with an increase in AIP, supporting this pathway (49, 50). However, in our cohort, the consistent association between RCA-FAI and MVCAD across glucose metabolism strata, even among patients with diabetes in whom the association between AIP and CAD is weakened, suggests that inflammation may operate independently or precede lipid dysregulation in severe CAD cases.

Our mediation analysis (27.9% indirect effect) supports a partially lipid-driven pathway but cannot rule out reverse causality or feedback loops. Prospective studies with serial FAI/AIP measurements are required to clarify temporal precedence.

If AIP primarily contributes to inflammation through lipid-driven mechanisms, intensive lipid-lowering therapy (such as statins or PCSK9 inhibitors, which can specifically reduce sdLDL-C levels) may help alleviate coronary artery inflammation (51, 52). Inflammation is assumed to be the main driving factor of dyslipidemia. In such cases, anti-inflammatory treatments (e.g., colchicine or canakinumab) can be used to prevent atherosclerosis (53, 54). The data from this study highlight the need to develop individualized treatment strategies based on a patient's lipid profile and inflammation levels.

The core value of this study lies in the fact that AIP and RCA-FAI could serve as clinically accessible alternative biomarkers for MetS. Although the direct quantification of sdLDL-C can provide mechanistic insights, this technique is technically demanding and costly, making its routine application difficult in most clinical settings. Similarly, although vascular inflammation is a key mechanism of atherosclerosis, direct histological assessment is invasive and cannot be used for routine risk stratification. In contrast, the AIP is calculated based on standard lipid components (TG and HDL-C) routinely measured in clinical practice at minimal additional cost. The RCA-FAI is derived from routine CCTA scans and calculated using established standardized radiomics protocols without the need for additional specialized imaging sequences or contrast agents. This makes AIP and RCA-FAI readily accessible as assessment indicators within existing cardiovascular diagnostic systems.

We confirmed a significant association between AIP and CAD severity, which was partially mediated by RCA-FAI. This provides clinicians with valuable and easily accessible routine screening tools, such as the lipid profile and CCTA. The dose–response relationship between RCA-FAI and CAD risk further underscores its potential value in risk assessment. Integrating these biomarkers can enhance the ability to identify individuals at high risk of MVCAD, overcoming the limitations of traditional risk factors and standard CCTA stenosis assessment. This easy-to-implement risk stratification can be used to guide more intensive preventive strategies (such as adjusting lipid-lowering therapy) or more frequent monitoring (such as regular CCTA follow-up), ultimately improving the prognosis of patients with MVCAD.

Computed tomography-derived fractional flow reserve (CT-FFR) enables the non-invasive assessment of the hemodynamic significance of coronary stenosis (9, 55). However, this study specifically excluded CT-FFR-based functional ischemia evaluation, focusing instead on pericoronary inflammation quantified via the right coronary artery fat attenuation index (RCA-FAI), given the distinct pathophysiological correlates (anatomical inflammation vs. hemodynamic impairment) of these two conditions. While lifelong endurance exercise has cardioprotective effects, recent evidence indicates that it does not improve coronary plaque composition compared with a standard healthy lifestyle (56). Exercise-mediated anti-inflammatory effects were deliberately excluded to prioritize the investigation of metabolic pathways.

### Study limitations and future directions

The principal strength of this study lies in being the first to systematically evaluate the relationship between AIP, RCA-FAI, and CAD severity across distinct glucose metabolism strata, with all CAD diagnoses confirmed using CCTA. However, several limitations warrant consideration. Causality constraints: The single-center cross-sectional design precludes the determination of causal relationships between RCA-FAI, AIP, and CAD progression. Sample size considerations: The moderate cohort size (*n* = 450) may limit the statistical power to detect subtle associations between RCA-FAI and AIP. Effect size interpretation: The modest regression coefficients observed between the RCA-FAI and AIP may reflect measurement variability rather than a true biological interaction. Confounding by pharmacotherapy: Chronic use of antihypertensives, hypoglycemics, statins, and aspirin—known to modulate lipid profiles, glycemic control, and vascular inflammation—could not be fully adjusted for in multivariable models. Surrogate marker limitations: Reliance on AIP as a proxy for sdLDL-C. Generalizability constraints: Exclusive enrolment of Chinese adults >45 years may have introduced selection bias, limiting extrapolation to younger populations or other ethnic groups. Future directions: Prospective large-scale multicenter randomized controlled trials are warranted to validate the RCA-FAI-mediated AIP–CAD pathway using sdLDL-C quantification and oxidative stress biomarkers, establish causality through longitudinal imaging pharmacological intervention studies, and explore ethnicity-specific variations in lipid-inflammation interactions.

## Conclusions

This study revealed a significant association between AIP and CAD severity in middle-aged and elderly Chinese populations, with RCA-FAI demonstrating a partial mediating effect on this relationship. These findings provide novel insights into the inflammatory mechanisms underlying CAD progression and highlight AIP's dual role of AIP as both a risk stratification tool and a potential therapeutic target. Future investigations should delineate the mechanistic interplay between sdLDL-C and CAD severity while validating AIP's clinical utility of AIP in personalized cardiovascular care.

## Data Availability

The data analyzed in this study are subject to the following licenses/restrictions: The study utilized existing clinical and imaging datasets from hospital records rather than prospectively collected data. Due to institutional privacy policies and patient confidentiality agreements, raw data cannot be shared upon request. Requests to access these datasets should be directed to LL at juanzi_0724@163.com.
